# The Role of Datasets on Scientific Influence within Conflict Research

**DOI:** 10.1371/journal.pone.0154148

**Published:** 2016-04-28

**Authors:** Tracy Van Holt, Jeffery C. Johnson, Shiloh Moates, Kathleen M. Carley

**Affiliations:** 1 Global Economic Dynamics and the Biosphere Program, Royal Swedish Academy of Sciences, Stockholm, Sweden; 2 Department of Anthropology, University of Florida, Gainesville, FL, United States of America; 3 Institute for Coastal Science and Policy, East Carolina University, Greenville, NC, United States of America; 4 Institute for Software Research, School of Computer Science, Carnegie Mellon University, Pittsburgh, PA, United States of America; Beihang University, CHINA

## Abstract

We inductively tested if a coherent field of inquiry in human conflict research emerged in an analysis of published research involving “conflict” in the Web of Science (WoS) over a 66-year period (1945–2011). We created a citation network that linked the 62,504 WoS records and their cited literature. We performed a critical path analysis (CPA), a specialized social network analysis on this citation network (~1.5 million works), to highlight the main contributions in conflict research and to test if research on conflict has in fact evolved to represent a coherent field of inquiry. Out of this vast dataset, 49 academic works were highlighted by the CPA suggesting a coherent field of inquiry; which means that researchers in the field acknowledge seminal contributions and share a common knowledge base. Other conflict concepts that were also analyzed—such as interpersonal conflict or conflict among pharmaceuticals, for example, did not form their own CP. A single path formed, meaning that there was a cohesive set of ideas that built upon previous research. This is in contrast to a main path analysis of conflict from 1957–1971 where ideas didn’t persist in that multiple paths existed and died or emerged reflecting lack of scientific coherence (Carley, Hummon, and Harty, 1993). The critical path consisted of a number of key features: 1) Concepts that built throughout include the notion that resource availability drives conflict, which emerged in the 1960s-1990s and continued on until 2011. More recent intrastate studies that focused on inequalities emerged from interstate studies on the democracy of peace earlier on the path. 2) Recent research on the path focused on forecasting conflict, which depends on well-developed metrics and theories to model. 3) We used keyword analysis to independently show how the CP was topically linked (i.e., through democracy, modeling, resources, and geography). Publically available conflict datasets developed early on helped shape the operationalization of conflict. In fact, 94% of the works on the CP that analyzed data either relied on publically available datasets, or they generated a dataset and made it public. These datasets appear to be important in the development of conflict research, allowing for cross-case comparisons, and comparisons to previous works.

## Introduction

In this article we seek to understand the emergence and evolution of a coherent scientific field of inquiry in the study of human conflict. We view the emergence of a coherent field of scientific research in terms of two important concepts in scientometrics: intellectual or knowledge base (the work of preceding scholars) and research front (the building of, the construction of new knowledge). This is not meant to be a comprehensive review of the literature on conflict research, but rather we analyze whether or not there is a path of scientific influence over an extended period of time. We let the data obtained from a Web of Science (WoS) search of “conflict” and the subsequent analysis of an extensive amount of data speak to the problem of interest, that being the presence or absence of an evolving science of conflict. The conflict literature reveals a number of competing arguments underlying the causes of conflict in human societies. But has this debate lead to a cohesive field of inquiry or has it produced a set of warring ideological factions? If so, what research forms the core of this field and what are the factors influencing a coherent body of work, if it exists? We compare our critical path analysis (CPA) to a similar analysis of conflict research published from 1957–1971 [1]. We also qualitatively and quantitatively analyze the critical path (CP) works in terms of topics, keywords, datasets used, authorship, and funding.

Bibliometrics have been widely used in sociology since the 1970's to analyze author citations of each other to characterize the structure of a discipline or impact of publications in a scientific field of endeavor [2,3]. There has also been an increasing general interest in visualizing and understanding the emergence of new scientific fields and the dynamics of scholarly collaboration [4–7]. Main path analysis (MPA) is an algorithm to identify the evolution of research. Each link in a MP represents the frequency with which article A is cited by article B in the citation history of all articles in the corpus. MPA identifies the citation path or paths from inception articles to the current articles that are the most "frequented" as new articles cite older articles. By tracing these weighted paths the development of theory can be seen in the dominant or main citation paths [1]. MPA makes clear the evolution of theory building in a particular research area over time and can pinpoint important contributions and divergences. More specifically, MPA involves the identification of source to sink vertices (i.e., articles) initially starting with the arc that has the greatest weight and subsequently moving at each step to the selection of arcs to neighbors with the greatest weight until the sink vertex is reached [8]. Thus the main path maps important or representative articles through time reflecting the "structural backbone" of the literature [8]. Carley, Hummon, and Harty [1] used MPA to reveal different leaders in the field than the simple traditional use of citation counts; this is because MPA measures scientific influence, rather than an author's popularity as measured by the number of citations per article. However, here we extend the analysis to find the critical path. CPA uses the main path to identify the path with the largest total sum of weights. So, whereas the MP may have more than one path from source articles to sink articles, CPA identifies that path that has the total greatest impact [8,9]. Thus a work is on the CP not because it cites many and is cited by many; but because it cites many who cited critical others and is cited by many who in turn are highly cited across all possible paths. Such works are a conduit for the flow of ideas and allow for the identification of the important theoretical and methodological topics in a corpus of work. CPA has been used in other fields including the science of social networks and clustering and classification science [8,10], among others.

Critical path analysis provides a few advantages over citation analysis alone: 1) It incorporates literature cited, which is how it identifies who cites each other and traces the evolution of a discipline whereas high impact author identifies only influential works. 2) Analyzing impact factors alone can identify influential works, but does not illustrate how these works are articulated in a broader scientific field of inquiry and research. Typically either author networks or citation networks are analyzed separately, and CPA integrates both. 3) Finally, CPA may include works without a full WoS record, such as books, if they appear in the literature cited section of a work. The CPA does not always include all popular authors because it is a measure of more than popularity alone. For example, Small and Singer’s 1976 “The War Proneness of Democratic Regimes” [11] is highly cited in the 1980s and early 1990s and is considered a seminal piece of literature in the debate of conflict and democracies though the paper did not appear on the path. Their work, however, is throughout the path since they originated the CoW dataset. While some key books did appear on the path, not all books did. Articles often cite books authored by Collier and Hoeffler [12,13] and Homer-Dixon [14] and game theory research cites Bueno de Mesquita [15] but these leading scholars did not appear. More traditional review methods or analyzes that are based on purely citations alone can help to tease apart noted scholars that are not on the path. Ethnic-conflict research draws from Horowitz’ book on ethnic groups in conflict [16], which explains many of the inequalities and ways to characterize ethnic groups, though the book doesn’t appear on the CP.

## Materials and Methods

First we selected all 62,504 records that were identified by a keyword search for “conflict” on the WoS from 1945 to 2011 in August 2011 from a library subscription that contained the complete records. We chose to select all research on conflict rather than a sub-discipline within the conflict literature because we were interested in the discipline as a whole and we didn’t want to inadvertently eliminate studies. For example, searching for specific terms such as “conflict, natural resource and war” resulted in 173 hits and “conflict, grazing, and Africa” only had 40 hits, which is likely because more abstract or complex concepts such as natural resources are difficult to isolate with a single search term [17]. By searching all research on “conflict” we included all areas of research—the field of psychology, interpersonal relations and even conflict of interacting medicines or of a biological pathway. For a record to be included, “conflict” appeared in the keyword, title, journal, or abstract fields. We included only original research articles and proceedings and eliminated review articles because we were interested in primary research. In Pajek, a software package for the analysis of large networks, we preprocessed these data to select which works were included and excluded in our analysis [9,18]. We removed any self-references and limited our analysis to only those works that were cited more than four times and to those articles that had citation lists according to recommendations by De Nooy, Mrvar and Batagelj [18]. Articles or books that are not indexed by WoS or those indexed that did not have citations included in WoS records were excluded.

We imported and processed these WoS records with WoSPajek (WoSPajek 0.7). This program parses the WoS records and extracts the title, authors, keywords, journal, publication year, and literature cited. The Monte Lingua extension standardizes the journal names [19]. WoSPajek produced a citation network, a matrix of works by works that is a matrix of the ties between the WoS records (works) and the works that cite that article (a work can include journal articles, books or other literature types); in our study, the citation network contained 1,510,243 works. We ran the CPA on the citation network in Pajek (version 2.03, 64 bit) (see S1 Table for full list of commands) [9]. The CP was constructed by including papers that have the highest centrality, both indegree (the number of citations an article receives) and outdegree (the number of cited references in the documents). CPA selects the works that are linked and have the highest weights until an end document is reached, the document is no longer cited, or there are no further works within the set [19,20].

Initially, we evaluated the 49 CP works by classifying how they were connected. This included a qualitative assessment of the themes that linked the works and an independent, quantitative keyword analysis. To qualitatively assess the linkages among works, we classified sections of the path based on the following themes: the role of democracy in interstate conflict, the need for relevant indicators, introducing key datasets that helped the discipline develop, and structural level analyses, availability theory (i.e. resource scarcity), and interstate conflict, which introduces ethnic and environmental conflict research. Spatially focused and forecasting analyses were also noted. For the keyword analysis, we parsed keywords from titles since keywords supplied by authors or WoS were not available for older journal articles. We generated a works by keywords network of the CP works and then visualized these keyword affiliation matrices. In these matrices two keywords are linked if works share the same keyword in the title. We eliminated words such as conflict or peace, which have ties to almost all articles, and obscure the structural relationship among keywords as well as country names or words like investigate that don’t help differentiate different types of analyses or thematic areas. To see which keywords were potentially most influential in terms of linking together other keywords, we sized the nodes of the keywords from the CP by betweeness centrality, which is a measure of the number of times a keyword lies on the shortest path between all other keywords.

Next we evaluated how our CP compared to 1) Carley, Hummon, and Harty’s [1] earlier study that traced the evolution of conflict literature in a single conflict journal and 2) a keyword analysis of all of the works in 2010 that we analyzed that were associated with conflict. Carley, Hummon, and Harty’s [1] MPA on articles from the Journal of Conflict Resolution (JCR) from (1957–1971) focused on JCR because the journal’s purpose was to develop the field of conflict science and they tested whether indicators of the evolution of a conflict science transpired. Today journal still is a leader in the field of conflict science, representing ~20 of the articles in our CP. One article from their original study also appeared in our CP. They did not analyze every article associated with “conflict” as we did, as this type of analysis was not possible when their paper was written. Their analysis, however, focused on similar topics and a pre-eminent conflict journal of the time. For the keyword visualization of 2010 articles, we isolated all of the articles from the WoS search for 2010 only, and used Pajek to link words together if keywords in titles co-occurred >59 times.

Finally, we quantified each of the 49 works in terms of the databases used, funding, associated workshops, conferences, joint projects, journals, and institutions. We then identified if the Correlates of War, Polity, or Armed Conflict datasets were used and noted if other databases were used or if the authors provided a way to access their data. We identified funding sources that were reported for each work, as well as if the authors thanked any particular workshops, conferences, or joint projects. We also identified the journals where the works were published and institutions of the authors. We identified whether the purpose of the article was to analyze data or not (theoretical articles, for example) and calculated if those articles that were data dependent, how many either used publically available datasets or made their own data publically available.

## Results and Discussion

### A cohesive field of inquiry

**Fig 1:**
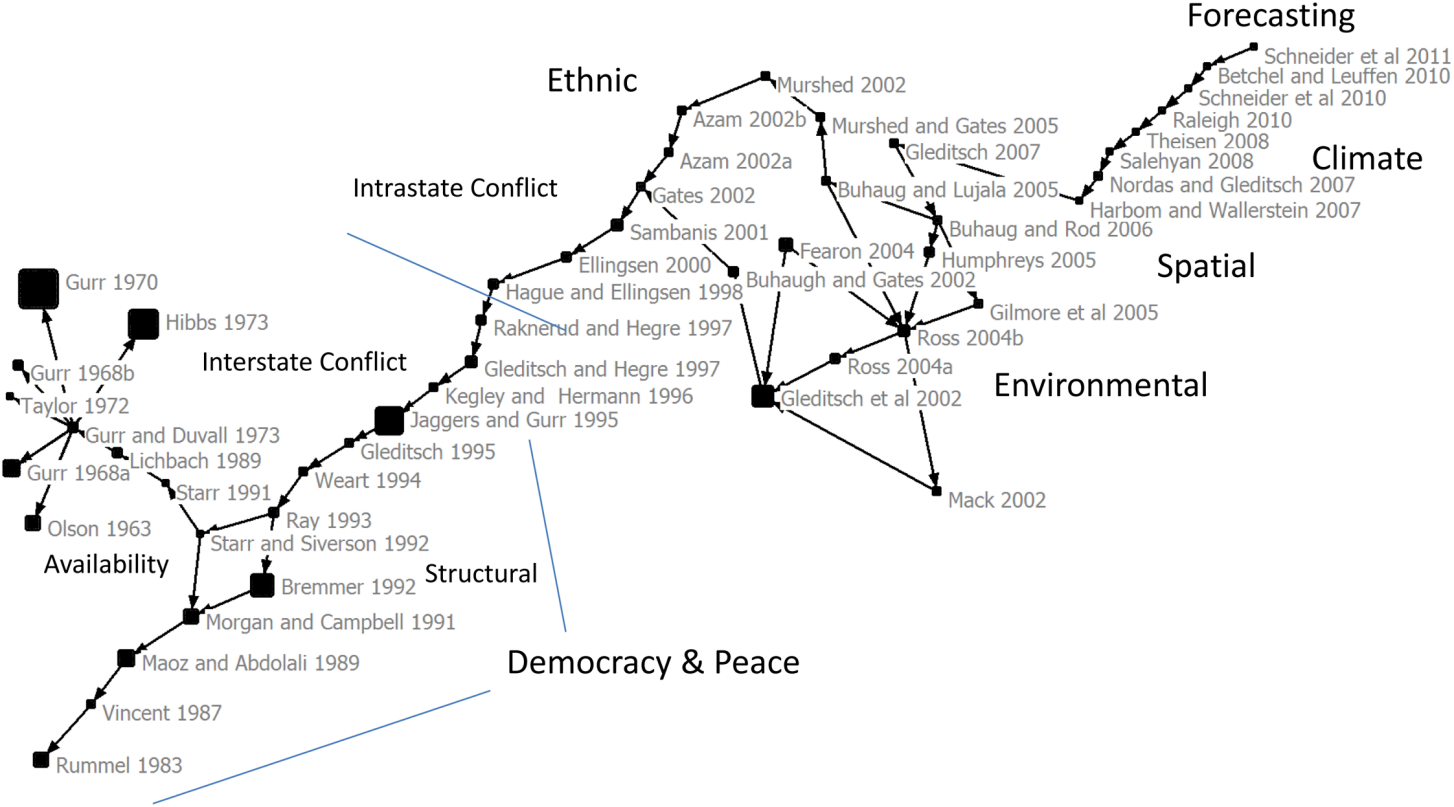
Critical path and scientific influence in conflict science. The critical path shows that conflict research was a coherent field of inquiry that built on the work of previous scholars. Earlier articles focused on the role of democracy in interstate conflict. They explained the need for relevant indicators, introduced key datasets that helped the discipline develop, and showed structural level analyses. The origins of availability theory, whether resource scarcity affects conflict, was the first node. Later articles focused on interstate conflict, which introduced ethic and environmental conflict research. Spatial analyses and forecasting built upon earlier indicators and theories to get finer resolution datasets and sophisticates analyses, which showed that the discipline was well developed. Node size was proportional to total citations as of 2011.

In order to construct a broad network of papers, we allowed for any research associated with “conflict” to appear in our dataset. The CP was obtained from a citation network of 1,510,243 works—derived from the 62,504 WoS records and cited literature of these works. The citation network was associated with 566,744 authors, 75,011 journals and 26,689 keywords. The CP showed a coherent field of inquiry that was represented by key influencing articles—49 articles in total—in conflict science that represented research from 1963–2011 (Fig 1). Our analysis of keyword topics in all “conflict” works in 2010 from the WoS (not only in the works highlighted by the CP) showed that other types of conflict were present, such as biomedical (i.e. drug conflicts), health, evolution, interpersonal, and organizational conflicts, in addition to governance, ethnic, environmental, and climate induced conflict (S1 Fig). These other areas of research did not form their own critical path or sections within our critical path, meaning that these other types of conflict did not form a cohesive field of inquiry in our analysis. The CP did not have any major branches and while there weren’t obvious factions, there was disagreement among authors about the drivers of conflict and research approaches; however, the works on the path built upon previous research and researchers framed their studies in relation to others, which suggests that the field of inquiry is cumulative.

Our finding is in contrast to Carley, Hummon, and Harty’s [1] earlier study of conflict science. Unfortunately, by 1971, they did not find any main paths or even a small set of main paths in the *Journal of Coflict Research* network; even when they created a meta-path, it was still unclear which were the main influencing articles [1]. According to Carley, Hummon, and Harty [1], “the dream of the movement’s founders, the emergence of a cross-disciplinary theory of conflcit, was not coming true”; however, we found evidence of a coherent field of inquiry that could be termed “conflict science”. In our analysis, we found evidence of a single critical path that indicated that this field had all the elements of a coherent scientific enterprise in terms of recognition of seminal works and research dynamics.

### Key research themes and approaches built upon previous work

The CP had a section focused on interstate conflict and an intrastate conflict. The intrastate section from the mid 1990’s onward contained research on environmental and ethnic conflicts and included spatial analyses and forecasting research, which depend on advanced theoretical models. This section was built upon the interstate research (1960’s to 1990’s) on democracy and peace where key conflict science theories, such as resource availability and democracy of peace, analyses and datasets were developed (Fig 1).

#### Resource availability research that developed from interstate conflict research linked to intrastate environmental research

*Availability concept developed*. Many debates in conflict studies that unfolded since the sixties challenge whether conflict was caused by resource scarcity or abundance; these early works focused on intrastate conflict. Olson [21] theorized that rapid economic growth—abundance—leads to political instability because class identity is lost, people migrate, and labor reorganizes. In contrast, Gurr [22] applied a concept from psychology and argued that when people perceived relative deprivation—a quality of life that falls short of their expectations—they revolt. Hibbs [23] did not find any evidence to support Gurr’s theory of relative deprivation as a cause for political violence in his study of the influence of racial, social, religious and cultural divisions on political violence. While Gurr and Duvall [24] addressed criticisms of the psychological origins of the concept of relative deprivation, this concept of availability (where scarcity and deprivation is the absence of availability) formed the foundation of much of conflict research, particularly that which focused on environmental conflict.

*Availability research focused on environmental resources*. Most articles from Hauge and Ellingsen [25] onward on the CP focused on interstate conflict and environmental issues. Harbom and Wallensteen [26] provided an armed conflict dataset showing that intrastate conflict (civil, ethnic, and environmental) accounted for recent conflict and interstate conflict had declined from 1946–2006. Their dataset showed that the rise in intrastate conflict research was likely a consequence of a true increase in this type of conflict more generally rather than an artifact of more scientific attention. Building on the concept of resource availability, Ross [27] evaluated 13 civil wars and natural resources and found that lootable resources—oil, minerals, and drugs—were most frequently associated with conflict and instrumental in extending conflicts [28]. Fearon’s [29] work confirmed that conflicts were longer when dominant ethnic groups and migrants fought over land or natural resources. In Humphreys [30] work linking conflict and agricultural commodities, he concluded that conflict was really about dependence, which is tightly linked to the resource availability argument, because when natural resources are threatened, actors try to end conflict based on past rather than future natural resource production.

*Resource availability linked to climate change*. The availability concept remained present in climate change-conflict research. Theisen [31] recreated Hague and Ellingsen’s earlier CP research [25] analyzing links between population density, soil degradation, deforestation, water scarcity, and civil war. Since poverty and dysfunctional institutions strongly explained civil conflict and land degradation was only weakly associated, Theisen concluded that the linkage between environmental scarcity and climate change was unlikely. Salehyan [32] called for more interdisciplinary research to understand the complex relationship between climate change and conflict. He argued that there was little consensus that climate change was a driver of conflict in part because climate-conflict research was environmentally deterministic because social and political variables had been excluded. Raleigh [33] addressed Salehyan’s concerns by including socio-political and environmental factors; Raleigh found that climate change exacerbated current tensions, rather than created conflict, with marginalized people more prone to environmental conflict. Nordas and Gleditsch [34] reiterated the need for global, dynamic, interdisciplinary models that operationalized intrastate conflict when the Intergovernmental Panel on Climate Change (IPCC) Third Assessment Report [35] found little evidence of climate change driving conflict.

#### Inequality concepts emerged in interstate democracy and peace research and linked to intrastate ethnic conflict research

*Democratic theory of peace evolved*. From Rummel [36] to Raknerud and Hegre [37], the CP works focused on the role of democracies in interstate conflict. Rummel [36] examined how elected leaders and representatives of libertarian states were subject to the will of the people when in conflict with other states. This formed the foundational work for the theory of democratic peace, that is, democracies lack conflict, which was present on the CP until the mid 1990’s. The debate about the democratic peace theory, which began in the Rummel-Vincent-Maoz-Morgan branch, continued as democracies evolved and research focused on when democracies *do* fight, which led to operationalizing inequalities. Ray [38] cited 19 instances of when cultural factors and structural constraints drove democratic states to fight each other. Weart [39] found 40 instances of major combat when oligarchies engaged in combat with democracies. Raknerud and Hegre [37] found that autocracies and democracies fight because they join each other’s wars. Kegley and Hermann [40] showed that democracies applied interventions instead of direct aggression. Gleditsch and Hegre [41] showed that young democracies were more prone to war compared to mature democracies because they had autocratic elements. As theory of democratic peace became more sophisticated, researchers moved away from whether democracies were involved in conflict or not and instead focused on the features of political systems that may indicate inequality, which tied in with the availability concept and laid the foundation for ethnic research at the intrastate level.

*Ethnic conflict measured system inequalities* Ellingsen [42] and Sambanis [43] marked the beginning of studies on the CP focusing on ethnic conflicts at the intrastate level, where, like the earlier democracy-peace studies, researchers focused on the features of the social system. For example, Ellingsen [42] classified degree of fragmentation of ethnic groups and the size of the largest minority and found that political regime and socio-economic characteristics were better predictors of domestic conflict. Sambanis [43] measured degree of ethnic fragmentation and integrated economic development indicators and found that ethnic wars were tied to political grievances, rather than economic opportunities. Gates [44] examined the role that ethnicity and ideology played in recruitment and allegiance during conflict. Azam [45] concluded that resources had to be redistributed for peace to emerge in his analysis of production capacity, productivity, and access to external funds in conflicts among two ethnic groups. In Murshed and Gates [46] fatalities in the Nepalese civil war was dependent on the human development index indicators and landlessness, which was related to ethnicity and caste.

#### Forecasting required advanced models that link to earlier path components

The most recent articles on the CP focused on forecasting conflict, which reflected the maturity of this field of inquiry [47–49]. For example to mine online news stories [50], run agent based models [51], or escalation games [52], researchers needed a theory to operationalize variables and test hypotheses. This is especially important in the social sciences, as theory development typically takes more time than in other sciences [53]. The structural, time series, and game theory forecasting approaches introduced by Schneider, Gleditsch and Carey [47] traced to earlier works on the CP analyzing the system structure. Indeed structural analyses have been an important element of conflict research since the early 1990’s [37,54–56] and game theory became popular, in part from research such as Azam and Hoeffler’s model of whether troops terrorized people to provide resources (i.e. food) or military assistance, which showed up earlier on the path [57].

### Keywords showed thematic linkages across CP articles

**Fig 2:**
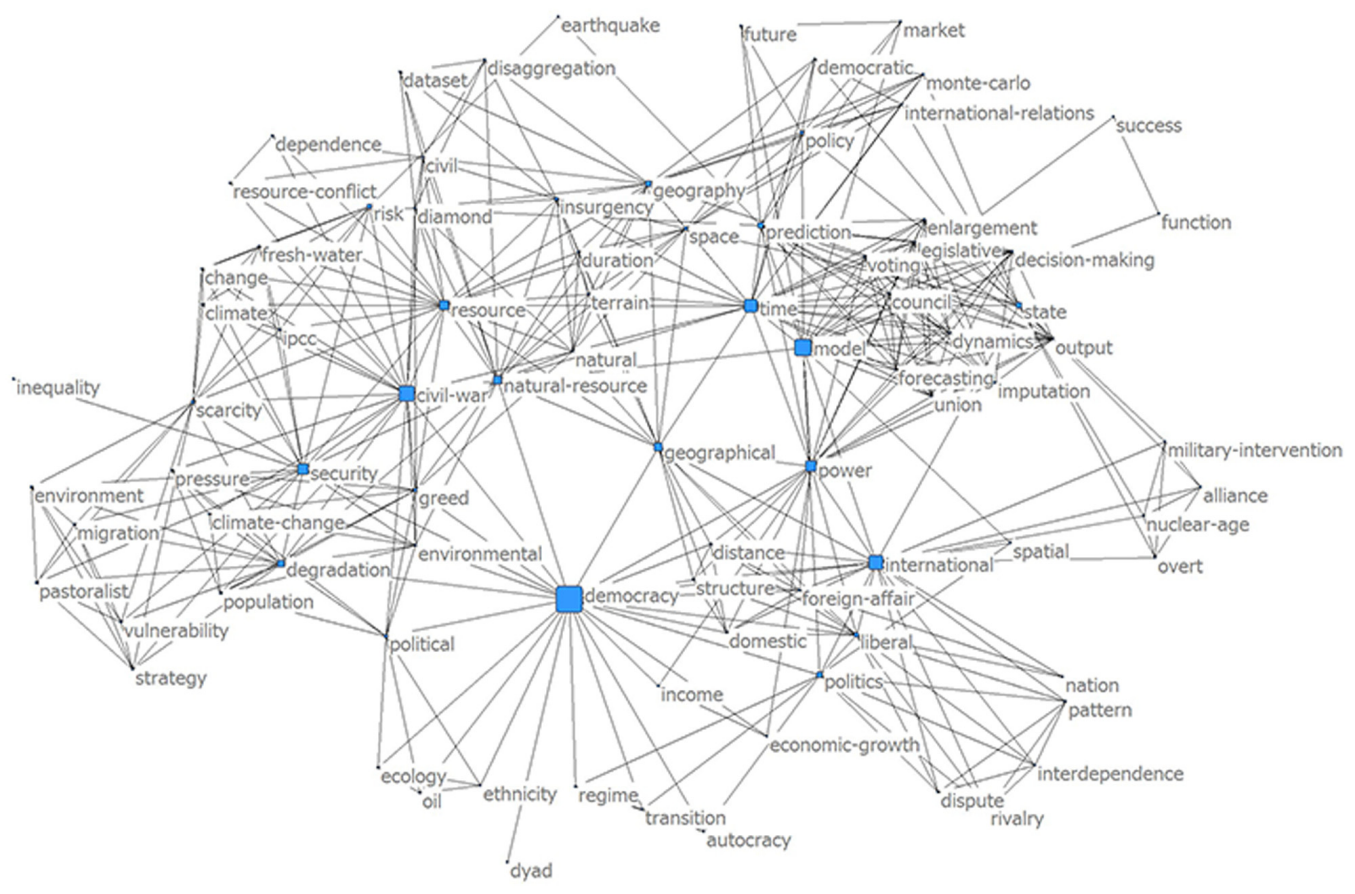
Keywords from the critical path conflict articles. Two keywords were connected if authors had the same pairs of keywords in the article title. Nodes were sized by the betweeness value, with higher betweeness indicating that the keyword was an imporant bridge across other keywords.

A link appears if two or more works on the CP shared the same keyword in the title (Fig 2). The high betweeness centrality value of *democracy*, *model*, *civil-war*, *time*, *power*, *international*, *resource*, *natural-resource*, and *security* indicated that these words were important thematic areas that linked research on the CP and was an independent indicator that the conflict field of inquiry built upon previous research. The lower portion of the figure represents interstate conflict. This included research focused on democracies (*democracy*) that represented research from the 1960–1990 and topics that directly emerged from that research, such as analyzing *structure*, *dyads*, *international*, *and foreign-affair*. Intrastate conflict (*civil war*), which was discussed from the late 1990’s onward, on the CP is located in the left portion of the figure, and was tightly linked to *resource/natural-resource* as well as *environmental*, *degradation*, *climate change*, *fresh-water*, and *resource-conflic*t. Here the word *scarcity*, which originated in the interstate conflict research, was fully embedded within the intrastate conflict work. The interdisciplinary areas of research (*geographical*) that depended on earlier theoretical advances had strong links to both the intra and interstate conflict areas. Recent conflict work focused on *forecasting*, *model*, and *prediction* that built upon topics from earlier works.

### Conflict datasets helped shape a coherent field of inquiry

The publically available datasets became an important factor in fostering a cohesive field of inquiry since everyone had access to these datasets; academics discussed both their strengths and limitations leading to the development of ever more sophisticated variables and analyses. For example, early on, Gurr and Duvall [24] discussed the need for relevant and robust indicators to more accurately depict conflict—“The indicators used to represent conceptual variables often are so partial and indirect, even dubious, that the results may mystify more than they confirm.” Gurr even critiqued his earlier work on the path for how he operationalized variables [22,58,59]. This transparency set the standard. Ninety-four percent of the works on the CP that analyzed data either relied on publically available datasets, or they generated datasets and made these public with 41% of the articles using more than one of these datasets. Considering all articles, even those that weren’t data dependent, the CoW dataset accounted for 45% of the articles, Armed Conflict Dataset 37%, Polity accounted for 32% or others 16%, and ~30% of the articles used more than one of these data types. When taking into account only the articles that actually depended on a dataset, the Correlates of War (CoW) data were the most widely used, accounting for 65% of the articles (see [25,28,31,36,60–62] for some examples). These data were important especially in the intrastate studies in the 1980’s and 1990’s on the CP. The CoW project began with Singer’s work [63] and originally measured only wars within the interstate system, although since 2010 they have added non-state and extra-state wars as well; today the 4.0 version of CoW still requires that armed forces are involved and a minimum threshold of 1,000 battle related fatalities (now within a 12 month timeframe)[64]; other related datasets are also available. The Armed Conflict dataset of the Uppsala/PRIO (Armed Conflict) data project [65,66] has been used by 53% of the CP articles (see [26,31,42,62,67,68] for some examples). This dataset provides a lower threshold of deaths (25) to identify conflicts where there is armed force and incompatibility between a government or territory or both, but at least one party is a state [65]. In twenty percent of the articles that used data, both the CoW and Armed Conflict datasets were used.

Data other than conflict measures were also needed. The polity persistence and change dataset [69–71], was most the widely used complimentary dataset used in 47% of the data-dependent articles (see [38,40,42,54,60,72–75] for some examples). Today Polity IV is available and this dataset classifies not only state polities according to their authority patterns but also takes into account anti-regime armed forces that challenge the definition of the polity itself (i.e. boundary disputes, etc). Earlier indictors used include Taylor and Hudson’s [76] handbook of indicators synthesizes UNESCO datasets and other data at the country level, such as International Labor Statistics, and number of protest demonstrations, which was a CP work. Intrastate and environmental conflict research called for finer resolution data that spans across larger areas. For example, in the past, FAO datasets have provided one value for a country on certain parameters [25,31] and more recently Buhuag combined the Armed Conflict dataset with forest and topographic information from FAO data and the World Conservation Monitoring Center of the UN Environment Program, as well as geographic features such as distance from the conflict center to the capital. While Azam [45] continued to use the Polity dataset, variables such as percentage of the country that is mountainous and magnitude of conflict area were also integrated. Thirty percent of the researchers on the path operationalized their own variables based on conflict theory and made these data public [27–29,45]. According to Humphreys [30], industry-wide datasets were a good source of data. The oil and diamond dataset presented by Humphreys [30] was derived from industry trade journals as well as the United States Department of Energy and the United States Geological Survey. Gilmore et al. on the CP [77] created *DIADATA*, which provided the geographic coordinates of diamond deposits. World Bank data on renewable and non-renewable resource production, the Center for International Earth Science Information Network at Columbia University, and Global Precipitation Climatology Project (GPCP) offer other potential sources.

The path was dominated by researchers affiliated with the Centre for the Study of Civil War, International Peace Research Institute, Oslo (PRIO) Norway (16 articles, about a third of the articles, were from the Center), nine articles were from Norwegian University of Science and Technology (NTNU), and four from the University of Oslo. Two journals dominated, a third of the articles were published in the Journal of Peace Research and 20% were in the Journal of Conflict Management, the journal that was the focus of our comparative analysis. The PRIO center set the standard in terms of collaborative research—they hosted scientists working in conflict from across the globe, housed datasets, and ran the Journal of Peace Research. US institutions appeared in key academic events that were regularly cited in the papers (40% of the articles). These included the Convention of the International Studies Association (3 papers) and the American Political Science Association (2 papers). Workshops included those held at Princeton University (2), the University of Chicago, Michigan State University, and the Workshop on Human Security and Climate Change in Norway. Funding came from a variety of sources with nine works funded by the Norwegian Research Council, eight funded by the World Bank, four by the Norwegian Ministry of Defense and by the Fridtjof Nansen Foundation for Science and the Humanities, and three by the National Science Foundation, the Ford Foundation and International Peace Research Institute (PRIO). What is clear is that in this coherent field of inquiry there are many opportunities to collaborate and connect with others, and working on similar datasets appears to be an influencing factor in the evolution of a conflict science.

## Conclusions

Our methodological approach to combine a broad keyword searches and critical path analysis, rather than an analysis within one journal or a simple citation analysis, is a promising way to identify scientific influence and key theoretical areas in a field. By casting our net wide, we illustrated how works were articulated in a broader scientific field of inquiry. Using a key word search instead of choosing for the contents of one journal, or of many journals, shed some lights on the structure of disciplines and how they transcend the journal domain. By characterizing the articles on the main path, we were able to identify that publically available datasets were key in forming this field of inquiry.

We finish by once again emphasizing this was not a comprehensive review of the conflict literature. What we have presented is a coherent field of inquiry that consists of 49 key articles that represented collaborations and shifts in the discipline from 1963 to 2011. There were a number of emerging themes and foci across the years often rising in response to the inadequacies of prior works’ theory, data, and analyses. What is clear is that there has been the development of a real focus on obtaining a more detailed understanding across a variety of temporal and spatial scales of the underlying causes of conflict across different contexts. This effort has been fueled by the need for access to relevant data, almost all of which were publically available, and the need for more sophisticated methods for valid measurement of important variables and the modeling of these data of ever improving quality. As more fine resolution data becomes available across the globe, scientists will be able to continue to develop unified conflict theories.

## Supporting Information

S1 Fig A keyword visualization of “conflict” articles from the Web of Science in 2010. In this visualization, words were linked if that keyword combination appeared >59 times. Topics included all aspects of conflict, including biomedical, health, evolution, interpersonal, and organizational, in addition to governance, ethnic, environmental, and climate induced conflict.
(PDF)

S1 Table Pajek Commands used to create the critical path. (PDF)
