# Supplementary figures and images for: The Role of Datasets on Scientific Influence within Conflict Research
Source: PLoS One. 2016 Apr 28;11(4):e0154148. doi: 10.1371/journal.pone.0154148 (PMC4849708; doi:10.1371/journal.pone.0154148)

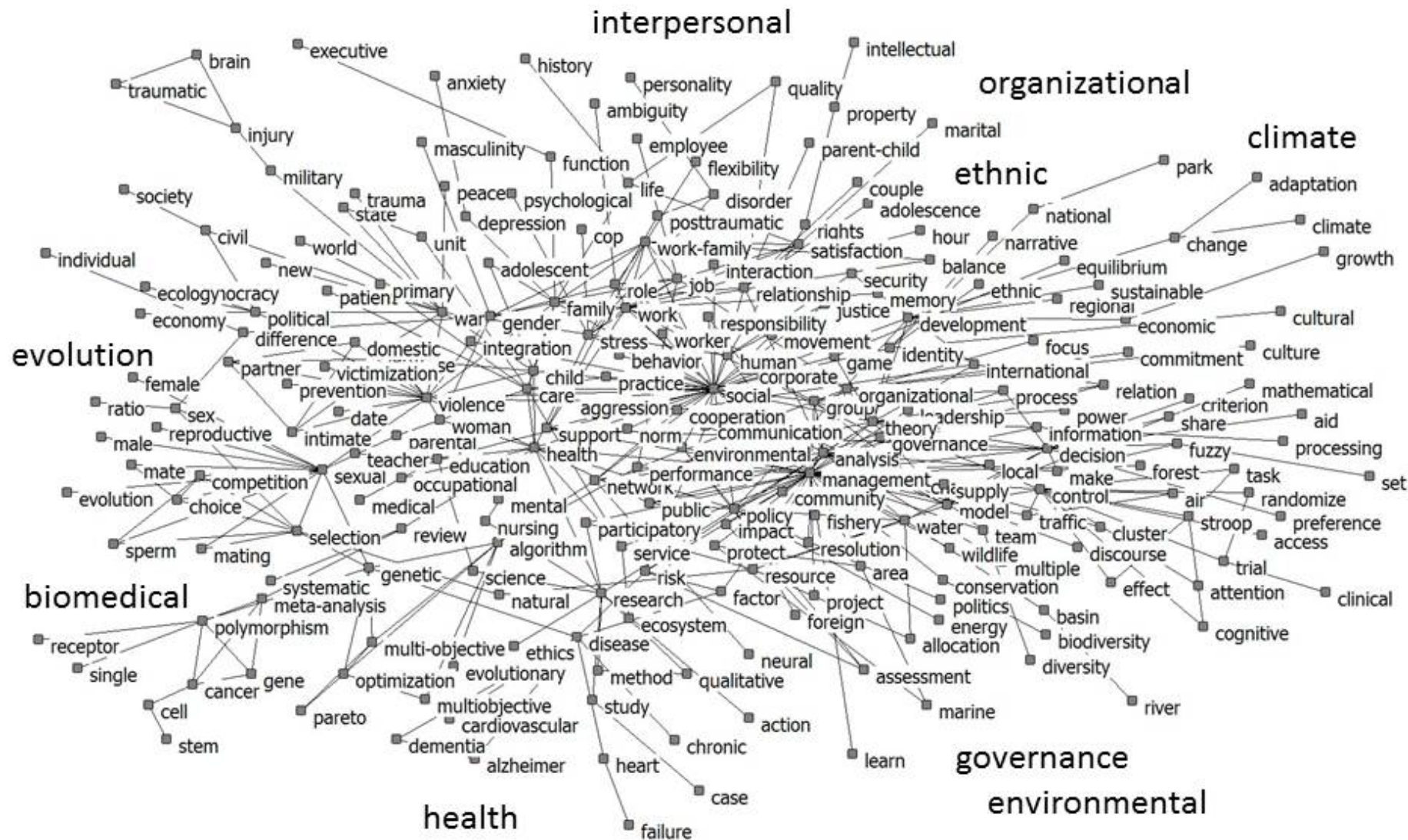

Supplement: S1 Fig — In this visualization, words were linked if that keyword combination appeared >59 times. Topics included all aspects of conflict, including biomedical, health, evolution, interpersonal, and organizational, in addition to governance, ethnic, environmental, and climate induced conflict. (PDF) [file pone.0154148.s001.pdf]
