# Supplementary material for: The Role of Datasets on Scientific Influence within Conflict Research
Source: PLoS One. 2016 Apr 28;11(4):e0154148. doi: 10.1371/journal.pone.0154148 (PMC4849708; doi:10.1371/journal.pone.0154148)
Supplement: S1 Table — (PDF) [file pone.0154148.s002.pdf]

**Pajek** version 2.03, 64 bit code	**Pajek 4.09, 64 bit code**
**Preprocessing:**	
Net/Transform/Remove/Loops	Network / Create New Network / Transform/Loops
Net/Transform/Remove lines/Multiple Lines/Single line	Network / Create New Network / Transform Remove lines/Multiple Lines/Single line
Net/Components/Strong [2]	Network / Create Partition / Components/Strong [2]
Operations/Shrink network/Partition [1] [0]	Operations / Network+Partition / Shrink Network/Partition [1] [0]
Net/ Transform/ Remove/Loops	Network / Create New Network / Transform/ Remove/Loops
**Creating the Network Boundary:**	
Net/Partition/Degree/Input	Network / Create Partition / Centrality / Degree
Partition/Binarize [1-(k-1)] (where k= works that are referenced at least k times)	Partition/Binarize [1-(k-1)] (where k= works that are referenced at least k times)
Net/Partition/Degree/Output	Network / Create Partition/ Centrality / Degree
Partition/Binarize [0]	Partition/Binarize 0
Partitions/Min (C1,C2) (make sure both partitions are selected first)	Partitions/Min (C1,C2) (make sure both partitions are selected first)
Operations/Extract from network/Partition [0]	Operations/ Network+Partition/ Extract Sub/Network
**Critical Path Analysis:**	
Nets/Critical Path Method (forget lines)	Network / Acyclic Network / Critical Path method - CPM
